# *p16^INK4a^* suppresses BRCA1-deficient mammary tumorigenesis

**DOI:** 10.18632/oncotarget.13015

**Published:** 2016-11-02

**Authors:** Alexandria Scott, Feng Bai, Ho Lam Chan, Shiqin Liu, Jinshan Ma, Joyce M Slingerland, David J. Robbins, Anthony J. Capobianco, Xin-Hai Pei

**Affiliations:** ^1^ Molecular Oncology Program, Department of Surgery, Miller School of Medicine, University of Miami, Miami, FL 33136, USA; ^2^ The Sheila and David Fuente Graduate Program in Cancer Biology, Miller School of Medicine, University of Miami, Miami, FL 33136, USA; ^3^ Braman Family Breast Cancer Institute, Sylvester Cancer Center, Miller School of Medicine, University of Miami, Miami, FL 33136, USA; ^4^ Sylvester Cancer Center, Miller School of Medicine, University of Miami, Miami, FL 33136, USA

**Keywords:** p16^INK4a^, senescence, brca1, breast cancer

## Abstract

Senescence prevents the proliferation of genomically damaged, but otherwise replication competent cells at risk of neoplastic transformation. p16^*INK4A*^ (p16), an inhibitor of CDK4 and CDK6, plays a critical role in controlling cellular senescence in multiple organs. Functional inactivation of p16 by gene mutation and promoter methylation is frequently detected in human breast cancers. However, deleting p16 in mice or targeting DNA methylation within the murine p16 promoter does not result in mammary tumorigenesis. How loss of p16 contributes to mammary tumorigenesis *in vivo* is not fully understood.

In this article, we reported that disruption of Brca1 in the mammary epithelium resulted in premature senescence that was rescued by p16 loss. We found that p16 loss transformed Brca1-deficient mammary epithelial cells and induced mammary tumors, though p16 loss alone was not sufficient to induce mammary tumorigenesis. We demonstrated that loss of both p16 and Brca1 led to metastatic, basal-like, mammary tumors with the induction of EMT and an enrichment of tumor initiating cells. We discovered that promoter methylation silenced *p16* expression in most of the tumors developed in mice heterozygous for p16 and lacking Brca1. These data not only identified the function of *p16* in suppressing BRCA1-deficient mammary tumorigenesis, but also revealed a collaborative effect of genetic mutation of p16 and epigenetic silencing of its transcription in promoting tumorigenesis. To the best of our knowledge, this is the first genetic evidence directly showing that p16 which is frequently deleted and inactivated in human breast cancers, collaborates with Brca1 controlling mammary tumorigenesis.

## INTRODUCTION

Senescence is a well-established barrier to tumorigenesis that prevents the proliferation of genomically damaged, but otherwise replication competent cells at risk of neoplastic transformation. Control of the G1 phase of the cell cycle is intrinsically linked with the maintenance of quiescent and senescent states in stem and somatic cells, respectively, and is primarily controlled by the INK4-CDK4/6-Rb pathway. The INK4 family of cell cycle inhibitors comprising of p16^*INK4A*^, p15^*INK4B*^, p18^*INK4C*^ and p19^*INK4D*^ (p16, p15, p18 and p19) inhibit CDK4 and CDK6, leading to the functional inactivation of RB [1]. Functional inactivation of this pathway is a common event in the development of most types of cancers [2], and also causes the loss of quiescent and senescent states of cells [3].

Unlike other INK4 family proteins, p16 is not expressed early, but is markedly increased with aging and senescence [4, 5]. Experiments in mice with a germline deletion of p16 reveal that p16 controls senescence in multiple organs during aging [6–9]. Importantly, p16 is deleted in ~50% of breast cancer cell lines, and p16 inactivation by DNA methylation occurs in ~30% of human breast cancers [2, 10, 11]. However, deleting p16 in mice or targeting DNA methylation within the murine p16 promoter does not result in mammary tumorigenesis but rather, these mice develop lymphomas and sarcomas with long latency [12–14]. How loss of p16 contributes to mammary tumorigenesis *in vivo* is not fully understood.

Breast cancer is heterogeneous with tumors that are both pathologically distinct and diverse in their responsiveness to treatment. Breast cancer is comprised of three main subtypes: HER2-positive, luminal, and basal-like cancers (BLBCs). Basal-like breast cancers (BLBCs) are ER-negative and more metastatic with few therapeutic options [15–18]. BRCA1 is a tumor suppressor, and its function has been linked with multiple pathways including DNA damage repair and oxidative stress regulation [19]. Functional loss of *BRCA1* by germline or somatic mutation or by promoter methylation is associated with more than one third of basal-like breast cancers and cell lines [20–22]. Patients with a BRCA1 deficiency develop BLBCs that are enriched with tumor-initiating cells (TICs) and exhibit epithelial-mesenchymal transition (EMT) characteristics [15, 16]. EMT, a process in which epithelial cells lose many of their epithelial characteristics and acquire mesenchymal features, plays an important role in tumor heterogeneity and generation of TICs [23]. TICs are thought to drive clinical relapse and metastasis [17, 18].

BRCA1 deficiency in human and mouse mammary epithelial cells activates both the p16-RB and p53 pathways, inducing premature senescence [24–26]. Consistently, heterozygous germline deletion of *Brca1* or specific deletion of *Brca1* in mouse mammary epithelial cells rarely develop mammary tumors. About 10% of *Brca1^+/−^* or *Brca1^f/f^*;MMTV-cre mice develop mammary tumors by 18 months of age [24, 25, 27–29]. Loss of p53 or its downstream target, p21, *in vivo* as well as knockdown of p16 or its downstream target, Rb, *in vitro* partially rescues the premature senescence of Brca1-deficient cells [24–28, 30], suggesting that disruption of the p53 and p16-Rb pathway are required to overcome Brca1-deficiency induced senescence and induce breast cancers. Indeed, inactivation of the p53 pathway enhances mammary tumor incidence and shortens the time of tumor onset in Brca1-deficient mice [27, 28, 31, 32]. Notably, most human BLBCs with functional loss of BRCA1 have dysfunctional p16-RB and p53 pathways [33–36]. However, most genetic studies of *Brca1* in mice co-mutate *Brca1* with one of the genes in the *p53* pathway [27, 28, 31, 32, 37]. It remains poorly understood whether p16 is involved in Brca1-deficient MEC senescence and tumorigenesis.

In this report, we generated *p16* and *Brca1* single and compound mutant mice to determine their function in controlling mammary epithelial cell (MEC) senescence and tumorigenesis.

## RESULTS

### p16 loss ameliorates Brca1 deficiency-induced senescence in MECs

**Figure 1:**
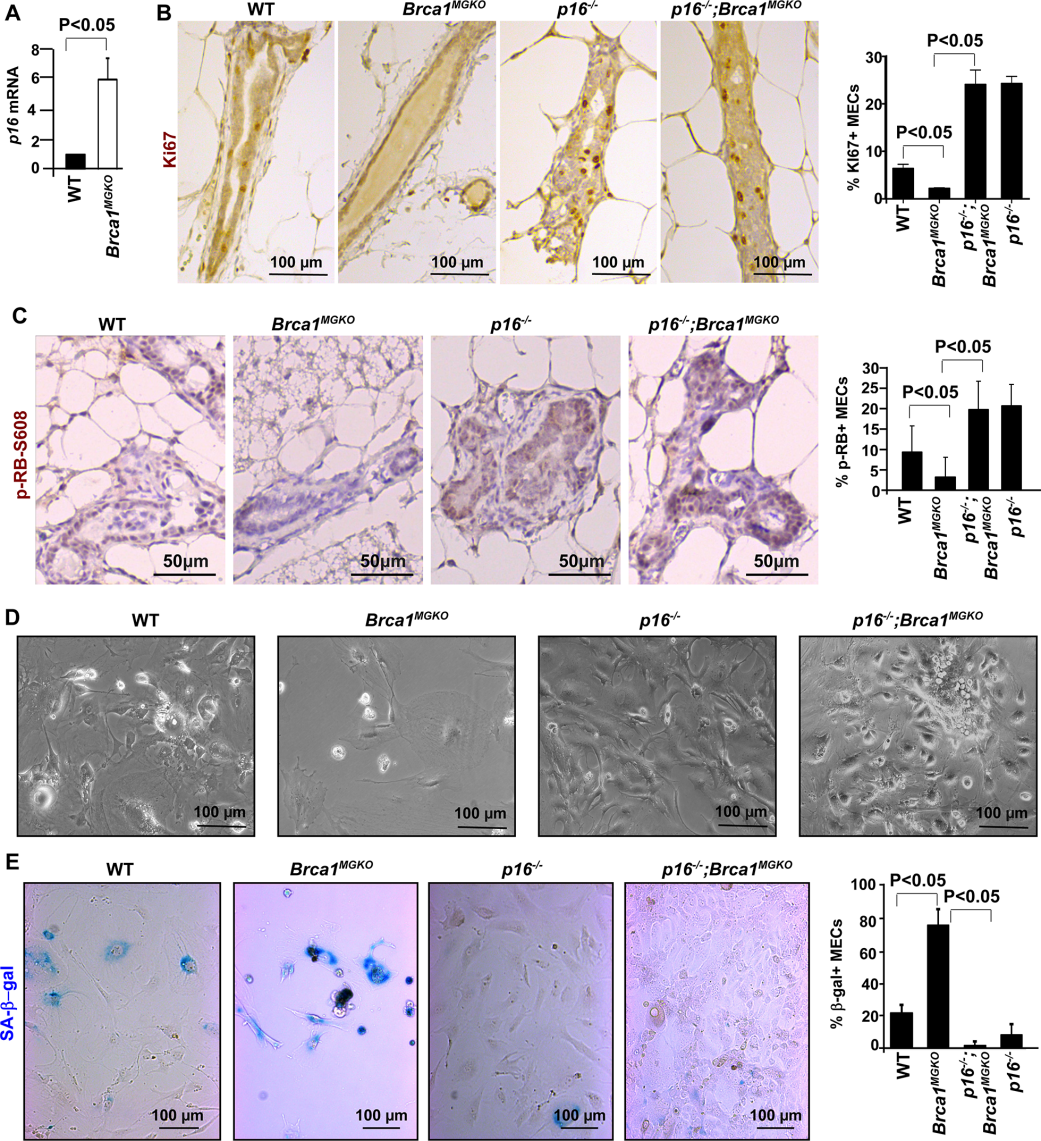
Loss of p16 rescues senescence caused by Brca1 deficiency (**A**) RT-QPCR of mammary tissue from age-matched WT and *Brca1^MGKO^* mice. Data represent the mean ± SD from triplicates of three mice per genotype. (**B**, **C**) Immunohistochemical staining of Ki67 and pRB-s608 in mammary glands of the indicated genotypes. Results represent the mean ± SD of three animals and two animals per group respectively. (**D**) MECs of the indicated genotypes were isolated and cultured to analyze cell morphology. (**E**) SA-β-gal assay of MECs of the indicated genotypes. Results represent the mean ± SD of triplicates per genotype.

We and others previously demonstrated that heterozygous germline mutation of Brca1 in human and mice leads to premature senescence in MECs [24, 26], though the molecular and cellular basis controlling Brca1-deficiency-induced senescence is not fully understood. To directly determine the role of Brca1 loss in mammary epithelial cell senescence and tumorigenesis, we used *Brca1^f/f^*;MMTV-Cre and *Brca1^f/^*^−^;MMTV-Cre mice (*Brca1^MGKO^*) mice as we previously described [25]. We compared *Brca1^MGKO^* mice to age-matched WT animals and found that mRNA levels of *p16* were increased in *Brca1^MGKO^* mammaries relative to WT counterparts (Figure 1A). Taking into consideration the growth defects and increased SA-β-gal positivity in *Brca1^MGKO^* MECs relative to WT counterparts (see below), these data suggest that Brca1-deficiency induced senescence in the mammary epithelium correlates with increased expression of *p16*.

Considering that Brca1 loss induces premature senescence with an increase of *p16* expression in MECs and that loss of p16 mitigates age-associated cellular senescence in compartments of the brain, pancreatic islets and blood [6–8], we were inspired to determine the role of p16 in controlling Brca1 deficiency-induced MEC senescence and tumorigenesis. To this end, we generated *p16^−/−^*;*Brca1^MGKO^* mice on a Balb/c-FVB-B6 mixed background. We found that the percentages of Ki67-positive MECs in *p16^−/−^* and *p16^−/−^;Brca1^MGKO^* mice (24.28 ± 2.1 and 24.06 ± 4.3) were significantly higher than those in WT mice (6.39 ± 1.3), which in turn were significantly higher than the percentage in *Brca1^MGKO^* (2.20 ± 0.2) mice (Figure 1B). We then directly examined Rb phosphorylation in MECs of different genotypes by using an antibody specifically recognizing Rb proteins phosphorylated at Ser608 by CDK4 and CDK6 [38, 39], the functional targets of p16. A consistent increase of pRb-Ser608 phosphorylation was detected in *p16^−/−^* mammary epithelia (9.86 ± 6.6% in WT to 21.64 ± 5.4% in *p16^−/−^*, Figure 1C), confirming the activation of CDK4 and/or CDK6. Consistent with our previous finding [24], 3.42 ± 5.0% pRb-Ser608 positive MECs were detected in *Brca1^MGKO^* females, which is significantly less than those in WT counterparts. However, *p16^−/−^;Brca1^MGKO^* mice had a significant higher percentage of positive MECs (20.69 ± 7.2%, Figure 1C) than *Brca1^MGKO^* mice. Together with the data derived from Ki67 staining, these results indicate that loss of p16 stimulates CDK4 and/or CDK6 activity toward the Rb protein in MECs, increasing their proliferation and rescuing the proliferative decline observed in Brca1-deficient MECs.

To further consolidate the role of loss of p16 and Brca1 in the regulation of MEC proliferation, we isolated and cultured primary MECs from virgin mice. We observed that *Brca1^MGKO^* MECs exhibited a large and flattened shape, a typical morphology of cellular senescence, while MECs from the other genotypes of mice were smaller and spindle-shaped (Figure 1D). Notably, most *Brca1^MGKO^* MECs exhibited strong, peri-nuclear staining of SA-β-gal whereas only a small population of WT MECs and very few *p16^−/−^* and *p16^−/−^;Brca1^MGKO^* MECs showed positive staining (Figure 1E). We then pulse-labeled primary MECs with bromodeoxyuridine (BrdU) for 15 hours and performed FACS analysis. *Brca1^MGKO^* MECs had increased G1 and decreased S phase cells relative to their WT counterparts (G1 phase cells, 29% vs 15%; S phase cells, 60% vs 68% Supplementary Figure S1). Importantly, MECs from *p16^−/−^* or *p16^−/−^;Brca1^MGKO^* mice displayed similar BrdU incorporation rates, 81% for *p16^−/−^* and 79% for *p16^−/^;Brca1^MGKO^*, which were significantly higher than their WT counterparts (Supplementary Figure S1). These data suggest that loss of *Brca1* induces senescence in MECs, which is rescued by p16 loss.

### Loss of p16 transforms Brca1-deficient MECs and induces mammary tumors

45% (*n* = 20) of *p16^−/−^* mice developed lymphoma and sarcoma in 24 months, which is consistent with previous reports [12, 13] (Table 1). Of the nine *p16^−/−^* tumors, two were detected in the mammary gland and were highly composed of lymphoma cells, as evidenced by their lymphocyte-like morphology, positivity for CD45 and CD31 by FACS analysis and negativity for Cdh1, an epithelial cell marker, by IHC (Supplementary Figure S2A, S2C and S2E). Interestingly, FACS analysis revealed that 1.7%–3.5% of the total tumor cell population was negative for CD45 and/or CD31 respectively (Supplementary Figure S2C), and IHC showed that less than 4% of sporadic tumor cells were epithelial-like and positively stained with Cdh1 (Supplementary Figure S2E), indicating that this tumor was comprised predominantly of lymphoma cells and that a small portion of cells originated from the mammary epithelium. These results confirm the predominant role of p16 in suppressing the development of lymphoma and sarcoma, and suggest that mammary tumorigenesis in p16 null mice may be masked by lymphomas and sarcomas.

**Table 1:** Spontaneous tumor development in WT and mutant female mice

Tumor	Wt	*p16^+/–^*	*p16^–/–^*	*Brca1^MGKO^^a^*	*p16^+/–^;Brca1^MGKO^*	*p16^–/–^;Brca1^MGKO^*
	11–24 m	11–24 m	11–24 m	11–24 m	11–24 m	11–24 m
Mammary Tumor	0/9^b^	0/6^c^	0/20^d^	0/8^e^	4/9^f^ (44%)	5/8^g^ (63%)
Metastasis					3/4^h^	2/5^i^
Ck14 + tumor					4/4	5/5
EMT + tumor					4/4	5/5
Other tumors	1/9^j^		9/20^k^	1/8^l^	3/9^m^	5/8^n^

a *Brca1^MGKO^*, *Brca1^f/−^;* MMTV-Cre or *Brca1^f/f^;* MMTV-Cre mice.

b one mouse was 24 months of age, and the remaining mice were 11–20 months of age.

c one mouse was 24 months of age, and the remaining mice were 11–20 months of age.

d two mice developed tumors in mammary glands composed of lymphoma cells and 1%–4.0% epithelial-like cells respectively.

e one mouse was 23 months of age, and the remaining mice were 11–19 months of age.

f four mice developed mammary tumors at 18, 18, 19 and 20 months of age, respectively. One mouse developed two different mammary tumors at two separate mammary glands. One mouse was 23 months of age, and the remaining mice were 14–20 months of age. One mouse was a breeder. Mammary tumor incidence, *p16^+/−^;Brca1^MGKO^* vs *p16^+/−^*, *P* = 0.103; *p16^+/−^; Brca1^MGKO^* vs *Brca1^MGKO^*, *P* = 0.082.

g five mice developed mammary tumors at 11, 12, 16, 17 and 20 months of age, respectively. Two mice developed two different mammary tumors at two separate mammary glands. One mouse was 20 months of age, and the remaining mice were 11–17 months of age. One mouse was a breeder. Mammary tumor incidence, *p16^−/−^; Brca1^MGKO^* vs *p16^−/−^*, *P* = 0.0006; *p16^−/−^;Brca1^MGKO^* vs *Brca1^MGKO^*, *P* = 0.026; *p16^mt^;Brca1^MGKO^* (*p16^+/−^ Brca1^MGKO^* and *p16^−/−^;Brca1^MGKO^*) vs *Brca1^MGKO^*, *P* = 0.022.

h lung metastasis from primary mammary tumors was detected in 3 mice, whose ages were 18, 18 and 20 months of age, respectively.

i lung metastasis from primary mammary tumors was detected in 2 mice, whose ages were 17 and 20 months of age, respectively.

j one mouse developed an ovarian tumor at 24 months of age.

k nine mice developed sarcoma or lymphoma.

l one mouse developed lymphoma at 15 months of age.

m three mice with mammary tumors also developed lymphomas.

n one mouse developed pancreatic carcinoma at 16 months of age, and three mice with mammary tumors also developed lymphoma, sarcoma, lung adenoma, and hepatocellular carcinoma respectively.

**Figure 2:**
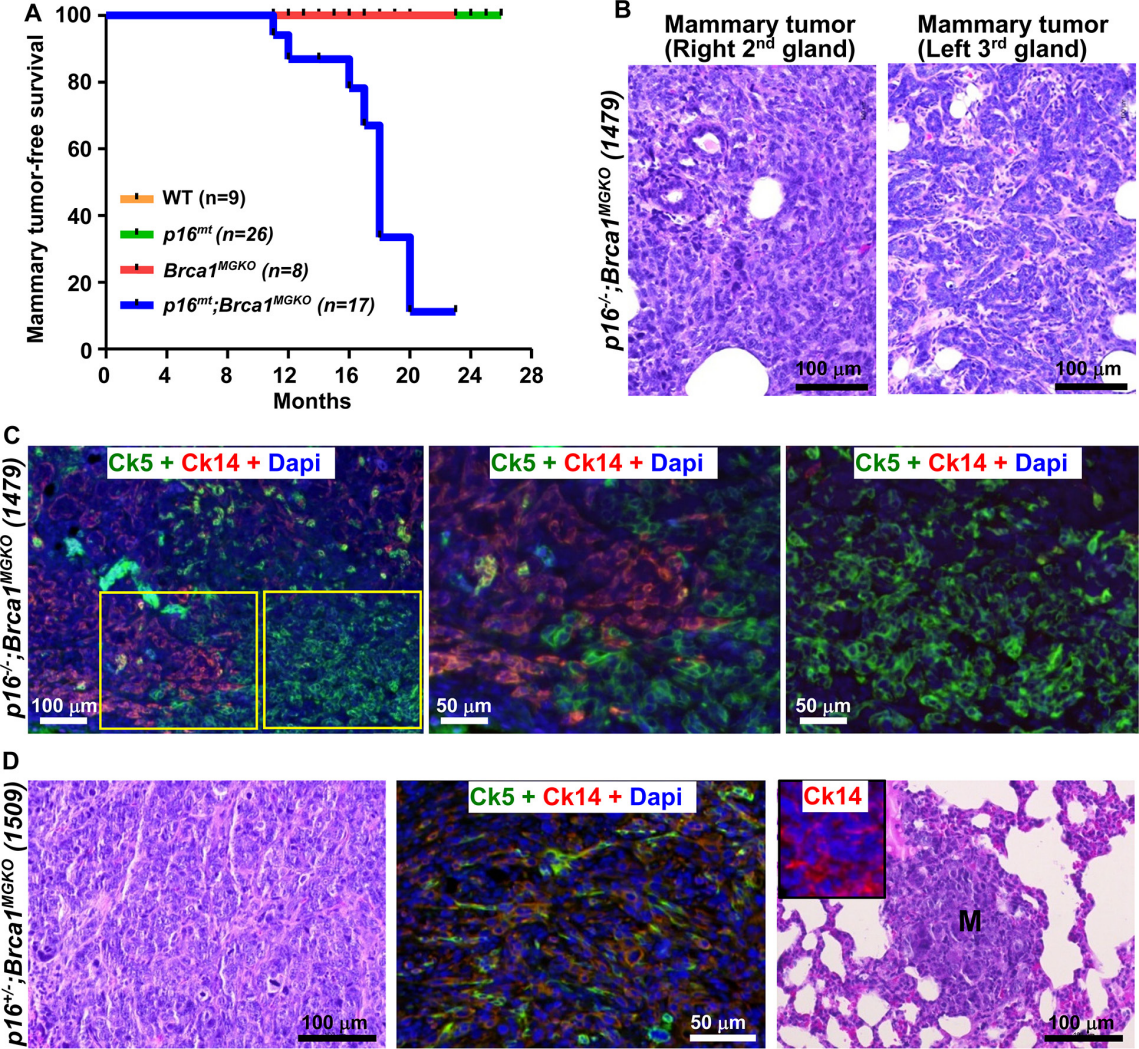
Characterization of primary tumors and distant metastases in mutant mice (**A**) Mammary tumor-free survival curve. *p16^mt^;Brca1^MGKO^* includes: *p16^+/−^;Brca1^MGKO^* and *p16^−/−^;Brca1^MGKO^* mice. *p16^mt^* includes: *p16^+/−^* and *p16^−/−^* mice. Log-rank (Mantel-cox) test: *p <* 0.0001 (**B**) H.E. staining of tumors in a *p16^−/−^;Brca1^MGKO^* mouse (1479) at 11 months of age. Note this mouse developed two different mammary tumors. (**C**) Immunofluorescence staining of mammary tumors in mouse 1479 with Ck14 and Ck5. The boxed areas in the left panel are enlarged in the middle and right panels. Note the majority of tumor cells are positive for either Ck5 or Ck14. (**D**) Immunofluorescence and H.E. staining of mammary tumor in a *p16^+/−^;Brca1^MGKO^* mouse (1509) at 18 months of age. Note this tumor was comprised predominately of Ck5 and Ck14+ cells (middle panel) and developed a distant metastasis (M) to the lung (right panel), which also comprised of Ck14+ cells (inset in the right panel).

We then followed the mammary tumor development in older mice and found that 63% of the *p16^−/−^;Brca1^MGKO^* mice (*n* = 8) and 44% of *p16^+/−^;Brca1^MGKO^* mice (*n* = 9) developed mammary tumors at 11–20 months and 16–23 months, respectively (Table 1, and Supplementary Table S1), whereas no *p16^−/−^*, *p16^+/−^* or *Brca1^MGKO^* mice did so at similar ages (Table 1). Median mammary tumor-free survival time in *p16^mt^;Brca1^MGKO^* mice (including *p16^−/−^;Brca1^MGKO^* and *p16^+/−^;Brca1^MGKO^* mice) was 18 months (Figure 2A). These results indicate that haploid or complete loss of p16 transforms Brca1-deficient mammary epithelial cells and induces mammary tumors.

### Depletion of both p16 and Brca1 leads to basal-like mammary tumors with EMT features

Mammary tumors developed in *p16^mt^;Brca1^MGKO^* mice were very aggressive and displayed typical morphological characteristics of highly malignant features (increased necrosis, spindle cells, nuclear-cytoplasm ratio, and mitotic indices) (Figure 2B, 2D, Supplementary Figure S2A, Supplementary Figure S3). 18% of the *p16^mt^;Brca1^MGKO^* mice (*n* = 17) developed two distinct mammary tumors in two separate mammary glands, demonstrating the ability of these mice to develop both intra- and inter- tumoral heterogeneity (Figure 2B, Supplementary Figure S3). Furthermore, 56% of the *p16^mt^;Brca1^MGKO^* mammary tumors (*n* = 9) metastasized to the lung in 17–20 months (Table 1, Figure 2D, Supplementary Table S1). All mammary tumors developed in *p16^mt^;Brca1^MGKO^* mice were positively stained with basal markers, Ck5 and Ck14 in 2–75% of total tumor cells (Figure 2C, 2D, Supplementary Figure S2B, Supplementary Figure S4, Supplementary Table S1). Further analysis revealed that most of these tumor cells expressed significantly reduced levels of Cdh1 protein when compared with levels in luminal cells of the mammary glands (Supplementary Figure S2E). Mammary tumors developed in *p16^mt^;Brca1^MGKO^* mice expressed 44% *Gata3*, and 54% *Elf5*, both of which are genes associated with luminal cell differentiation, relative to the tumor-free mammary tissues of the same mouse (Supplementary Figure S2D), confirming Brca1-deficiency impaired luminal differentiation during tumorigenesis, as we previously demonstrated [24, 25]. All mammary tumors derived from *p16^mt^;Brca1^MGKO^* mice were stained positively for vimentin (Vim), a mesenchymal marker, and Twist, an EMT transcription factor (Table 1, Supplementary Figure S2F). These data indicate that depletion of both p16 and Brca1 results in basal-like mammary tumors with activation of EMT, which is consistent with our previous finding that deletion of *Brca1* activates EMT in mammary tumorigenesis [25].

We screened 43 human invasive breast cancers and selected 10 ER-negative samples with the lowest *BRCA1* mRNA expression as previously described [25]. We compared tumor pathology and expression of CK5 and CK14 in these samples with mouse mammary tumors. We noticed that both the tumor cell morphology and expression pattern of CK5 and CK14 in *p16;Brca1* double mutant mouse mammary tumors resembled human basal-like breast cancers that were ER-negative and expressed low BRCA1 (Supplementary Figure S4).

Given the function of Brca1 in DNA damage repair, we also evaluated the role of Brca1 loss in inducing DNA damage in tumor development. We determined the expression of γH2AX, a marker for DNA double-strand breaks, in spontaneous tumors from mutant mice. Since *p16* single-mutant mice only developed lymphoma and sarcoma, we compared γH2AX expression in these tumors with mammary tumors. We found that the number of γH2AX-positive cells in *p16^mt^;Brca1^MGKO^* tumors was significantly greater than in *p16* single-mutant tumors (4.5% ± 2.5% vs 0.84% ± 0.36%, *p <* 0.05, Supplementary Figure S2G, S2H), indicating a significant increase of cells with DNA damage in *Brca1*-deficient tumors.

Together, these results suggest that depletion of both p16 and Brca1 induces metastatic basal-like tumors that have an activated EMT program and enhanced DNA damage.

### *p16 and Brca1* double-mutant tumor cells are transplantable

**Figure 3:**
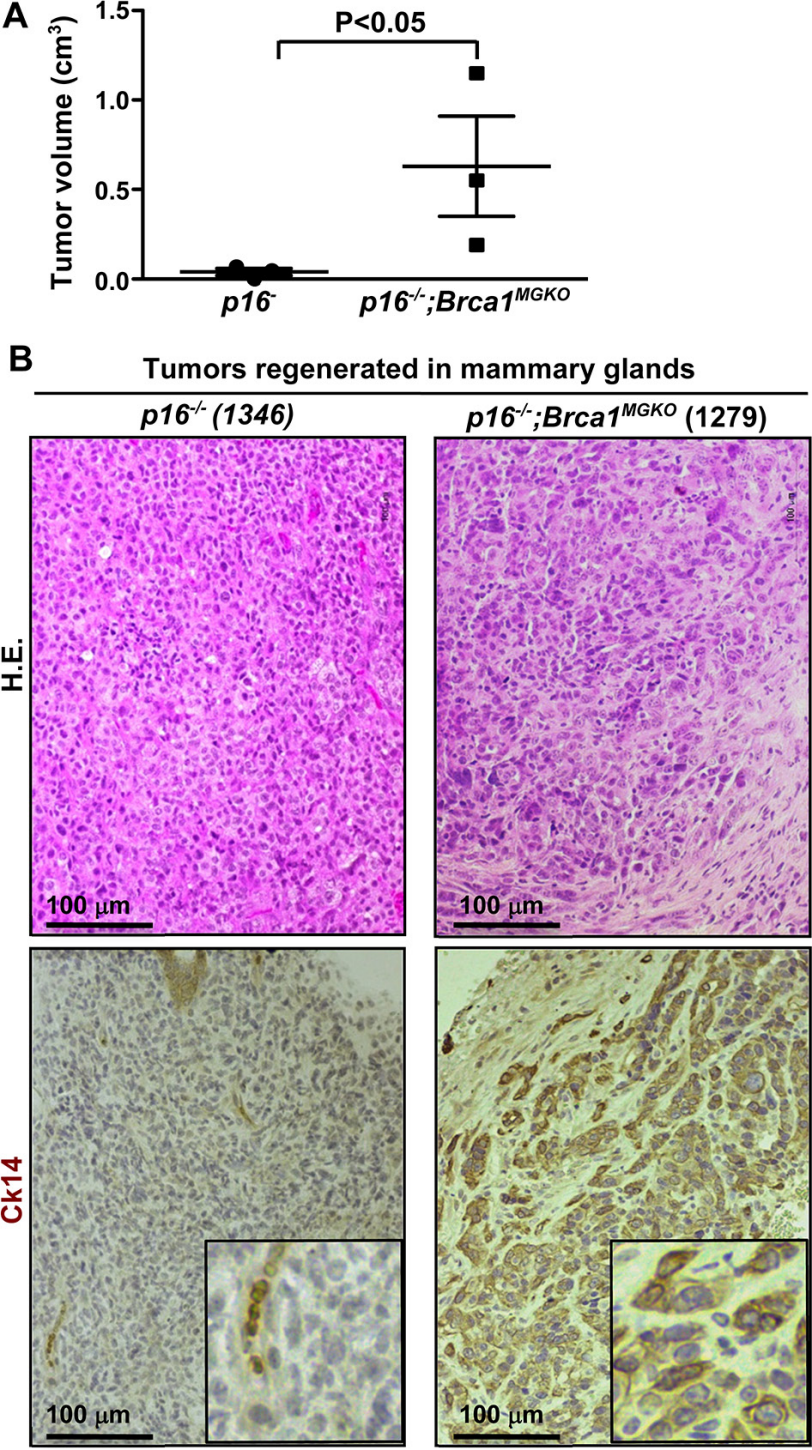
*p16;Brca1* compound mutant tumor cells are enriched with TICs and capable of generating basal-like mammary tumors (**A**) 1 × 10^6^ cells from the tumors developed in the mammary glands of *p16^−/−^* and *p16^−/−^;Brca1^MGKO^* mice were transplanted into the left and right inguinal MFPs of female NSG mice, respectively. Generated tumor volume was measured 4 weeks after transplant. Results are representative of the mean ± SD of three tumors of each genotype. (**B**) Representative H.E. and immunostaining of Ck14 of regenerated mammary tumors.

A small portion of epithelial-like cells observed in two primary *p16^−/−^* lymphomas that developed in mammary glands were sporadic and cuboid, luminal-like cells that were negative for Ck14 and positive for Cdh1 (Supplementary Figure S2B and S2E). We transplanted 1 × 10^6^ cells from one of, and 4 × 10^6^ cells from the other one of the two *p16^−/−^* tumors that are mixed with lymphoma and epithelial-like cells into MFPs of NSG mice (three recipients per primary tumor). Tumors regenerated, like *p16^−/−^* primary tumors, were predominantly composed of lymphoma cells mixed with Cdh1-positive and Ck14-negative tumor cells (Figure 3, Supplementary Figure S2B, S2I, and data not shown). The Cdh1-positive cells in the regenerated tumors accounted for < 5% of total tumor cells, however, they aggregated together and formed a larger mass of epithelial-like tissue than in primary tumors (Supplementary Figure S2I and S2E). This result suggests that p16 loss stimulates luminal epithelial cell proliferation, possibly contributing to the development of premalignant lesions. These data are also consistent with our finding that loss of p18, another Ink4 family cell cycle inhibitor, promotes luminal epithelial cell proliferation and induces luminal tumorigenesis [40].

We transplanted 1 × 10^6^ cells from a *p16^−/−^; Brca1^MGKO^* mammary tumor, and 4 × 10^6^ cells from a *p16^+/−^; Brca1^MGKO^* mammary tumor, respectively, into MFPs of NSG mice (three recipients per primary tumor). We found that *p16^mt^;Brca1^MGKO^* tumor cells regenerated significantly larger tumors than *p16^−/−^* cells with the same number of cells in the same time period (Figure 3A, and data not shown). Further analysis revealed that the generated *p16^mt^;Brca1^MGKO^* tumors resembled the pathology of the primary tumors and were positive for Ck14 recapitulating the phenotype observed in primary mammary tumors (Figure 3B). These data suggest that *p16;Brca1* double-mutant mammary tumors are enriched with mammary tumor initiating cells.

### Expression of *p16* is lost in *p16^+/–^*;*Brca1^MGKO^* tumors due to promoter methylation

**Figure 4:**
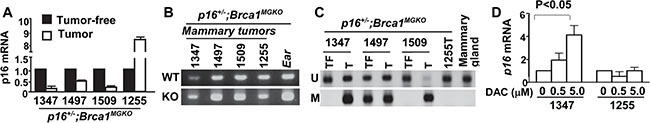
Promoter methylation silences *p16* expression in *p16^+/–^;Brca1^MGKO^* tumors (**A**) mRNA expression of *p16* in *p16^+/−^; Brca1^MGKO^* mammary tumors was determined by q-RT-PCR. Corresponding tumor-free mammary tissues from the same mice were used as controls. Note the control mammary tissue for mouse 1497 tumor was from a premalignant lesion of the same mouse. Data are expressed as the mean of triplicate experiments. (**B**) LOH analysis of the *p16* gene in *p16^+/−^;Brca1^MGKO^* mammary tumors. Ear DNA was used as a control. (**C**) Bisulphite-treated DNA from mammary tumors (T) or tumor-free mammary tissues (TF) from the same mice was analyzed for methylation of p16. U, unmethylated. M, methylated. A normal mammary gland from a WT mouse was used as a control. Note, no methylated p16 was detected in the mammary tumor developed in mouse 1255 in which the *p16* mRNA level was not reduced relative to tumor-free mammary tissue of the same mouse. (**D**) *p16* mRNA analysis in primary mammary tumor cells after treatment with DAC at 0, 0.5, or 5 μM for 72 hours.

We determined the expression of *p16* in *p16^+/−^; Brca1^MGKO^* mammary tumors. We found that *p16* mRNA expression was retained in one tumor (number 1255), but clearly reduced in the other tumors when compared with tumor-free mammary tissues from the same mouse (Figure 4A). *p16* mRNA levels in tumor 1255 were 8.5-fold more than tumor-free mammary tissues, which could be caused by a reduction in the function of the Rb or p53 pathways [41, 42]. We performed loss of heterozygosity (LOH) analysis for DNA extracted from *p16^+/−^;Brca1^MGKO^* mammary tumors and found that the remaining WT allele of *p16* was retained in all four tumors (Figure 4B). These results suggest that epigenetic silencing of *p16*, likely by promoter methylation as observed in DMBA-induced *p16^+/−^* lymphomas and sarcomas [12], plays an important role in *p16^+/−^;Brca1^MGKO^* mammary tumorigenesis. To determine the methylation status of the *p16^+/−^;Brca1^MGKO^* mammary tumors, we performed methylation-specific PCR (MS-PCR) and detected methylation of the *p16* promoter in three tumors with reduced expression of *p16*, but not in tumor 1255 in which *p16* expression was not decreased (Figure 4C). Notably, we also detected *p16* promoter methylation in a tumor-free, but premalignant lesion-containing mammary tissue in the mouse 1497 (Figure 4C), which explains why there was not a significant decrease in *p16* expression between tumor vs tumor-free tissues in mouse 1497 (Figure 4A). We isolated and cultured primary cells from *p16^+/−^;Brca1^MGKO^* mammary tumors and found that *p16* mRNA levels were significantly increased in the 1347 tumor cells treated with 5-aza-2′-deoxycytidine (DAC), a methylation inhibitor, but not in the 1255 tumor cells (Figure 4D), confirming the findings derived by MS-PCR in tumors. These results suggest that epigenetic silencing of p16 by promoter methylation plays an important role in the development of, at least, some of the *p16^+/−^;Brca1^MGKO^* mammary tumors.

## DISCUSSION

In this article, we reported that disruption of Brca1 in the mammary epithelium results in premature senescence with an increase of p*16* expression, which is rescued by p16 loss. We found that loss of p16 transforms Brca1-deficient mammary epithelial cells and induces mammary tumors, though p16 loss alone is not sufficient to induce spontaneous mammary tumorigenesis. We showed that mammary tumors deficient for both p16 and Brca1 are highly aggressive, metastatic, and enriched for TICs. We demonstrated that loss of p16 and Brca1 collaboratively induce basal-like mammary tumor development with the induction of EMT. To the best of our knowledge, this is the first genetic evidence directly showing that p16 which is frequently deleted and inactivated in human breast cancers, collaborates with Brca1 controlling mammary tumorigenesis.

The functions of BRCA1 have been linked with multiple pathways. BRCA1 deficiency causes chromosomal abnormalities, leading to the activation of DNA-damage checkpoint pathways and premature senescence [26–28, 30]. It has also been reported that BRCA1 deficiency in MECs impairs stability and activation of Nrf2, a key transcription factor in regulating antioxidant response, and leads to the accumulation of reactive oxygen species (ROS), along with the increase of p16 [43–45]. Interestingly, accumulation of ROS has been associated with cellular senescence [46], and Nrf2 activation restores ROS levels in Brca1-deficient MECs [43, 44]. These findings suggest that BRCA1 deficiency induced premature senescence in MECs, at least, partially resulted from accumulation of ROS. Our results that loss of p16 rescues the MEC senescence caused by Brca1 deficiency suggests that p16 loss may allow Nrf2 levels to accumulate in Brca1-deficient cells and suppress ROS, which remains to be determined.

The finding that loss of p16 rescues MEC senescence caused by Brca1 deficiency also suggests that p16 blocks these cells from entering an active cell cycle. These results indicate that, in addition to the p53-p21 pathway that is activated by BRCA1 loss [27, 28, 30], p16 is also a critical downstream target of BRCA1 in controlling mammary cell proliferation and senescence. In line with these data, it was recently reported *in vitro* that BRCA1 knockdown enhances the association of BRG1, a chromatin-remodeling factor that interacts with BRCA1, with the promoters of p16 and p21, leading to activation of their transcription and senescence [30]. More recently, it was found that human mammary epithelial cells from BRCA1-mutation carriers exhibit senescence, which is triggered by pRb pathway activation [26]. Since deregulation of p53 alone induces DNA damage, *p16; Brca1* compound mutant mice and cells offer a unique opportunity to investigate the role of Brca1 in DNA damage repair under a genetically p53 intact background.

The function of p16 in breast cancer suppression has been extensively studied and confirmed in human breast cancer samples and cell lines [2, 47]. However, the role of p16 in suppressing mammary tumorigenesis *in vivo* is elusive. Previous findings [12–14] that mice lacking p16 or targeting DNA methylation within the *p16* promoter rarely develop mammary tumors suggests that p16 loss alone is not sufficient for mammary tumorigenesis *in vivo.* Interestingly, loss of p16 increases MEC proliferation, rescues Brca1-deficiency induced MEC senescence, and induces mammary tumors in a Brca1-deficient background, suggesting that p16 collaborates with Brca1 to suppress mammary tumorigenesis. Though the remaining WT allele of *p16* was retained in all *p16^+/−^; Brca1*^MGKO^ mammary tumors, the *p16* promoter was methylated and *p16* mRNA was significantly reduced in most of these tumors, indicating that silencing of p16 by promoter methylation plays a role in the development of, at least, some of the *p16^+/−^;Brca1*^MGKO^ mammary tumors. In line with these results are the clinical findings that methylation of BRCA1 and p16 is frequently detected in sporadic breast cancers and has a predictive value for tumor recurrence [48]. Together, our data not only support the function of p16 in suppression of Brca1-deficient mammary tumorigenesis, but also indicate that genetic mutation of p16 cooperates with epigenetic silencing of its transcription to promote tumorigenesis.

Of the four INK4 genes, p16 is frequently deleted and inactivated, and p18 expression is significantly downregulated in breast cancers ([1, 2, 10, 11, 34], and Pei XH, unpublished). The Rb family proteins consist of RB, p107, and p130, which are also frequently deleted and inactivated in breast cancers and are downstream targets of *INK4* proteins [1, 2, 10, 11, 34]. RB is a major target for genomic disruption in *BRCA1* mutant human breast cancers and loss of both RB and BRCA1 is a feature of basal-like breast cancers [34, 36, 49]. Deletion of both Rb and p107 in mouse epithelia results in mammary luminal tumor development [49]. We demonstrated that deletion of *p18* in mice stimulates luminal progenitor cell proliferation, leading to mammary luminal tumorigenesis [40], and that depletion of Brca1 in p18 null mice converts luminal tumors into basal like tumors and activates EMT [24, 25]. We observed that depletion of p19 also stimulates mammary luminal cell proliferation [50]. In the present study, we report that loss of p16 increases MEC proliferation and induces mammary tumorigenesis in a Brca1- deficient background. More importantly, all *p16;Brca1* compound mutant mammary tumors are poorly differentiated basal-like tumors with enriched TICs and activated EMT features. These findings suggest that the INK4-Rb pathway suppresses mammary luminal cell proliferation and tumorigenesis, and collaborates with Brca1 to control basal-like tumorigenesis and EMT.

## MATERIALS AND METHODS

### Mice

The generation of *Brca1^f/f^*;MMTV-Cre and *Brca1^f/–^*; MMTV-Cre mice in a Balb/c-B6 mixed background has been described previously [25]. Virgin *Brca1^f/f^*;MMTV-Cre and *Brca1^f/–^*;MMTV-Cre (*Brca1^MGKO^*) mammaries express < 20% of Brca1 protein and mRNA relative to the levels in *Brca1^f/+^*;MMTV-Cre, indicating depletion of Brca1 in the mammary epithelia [25]. *p16* mutant mice in a FVB background were gifted by our collaborators, Dr. Norman Sharpless [12]. Virgin female mice were used in the study unless otherwise specified. All animal procedures were approved by the Institutional Animal Care and Use Committee at the University of North Carolina and University of Miami.

### Histopathology, IHC, and qRT-PCR

Histopathology and IHC were performed as previously described [24, 25]. Primary antibodies used are as follows: Ki67 (Novocastra Laboratories, Newcastle upon Tyne, UK), gH2AX, phospho-RB (Cell Signaling), CK14 (Thermo Scientific), ERα, (Santa Cruz), Vim, Twist (Abcam), Cdh1 (BD Biosciences), CK5 (Covance prb-160p). Mammary tumors in which at least two EMT markers (decreased Cdh1, increased Vim or Twist) were detected in > 2% tumor cells are defined as EMT+ tumors, as we previously described [25]. The IACUC (Institutional Animal Care and Use Committee) at the University of Miami approved all procedures. QRT-PCR was carried out as previously described [24, 25].

### Mammary cell preparation, cell cycle analysis, and mammary tumor cell transplantation

Mammary glands were dissected from virgin female mice at the indicated ages and genotypes. After mechanical dissociation, the tissue was processed as previously described [24, 25, 40]. MECs isolated from mice were cultured in in DMEM, with 10% FBS, 10 μg/ml insulin, 10 ng/ml EGF, 10 μg/ml Hydrocortisone. For cell cycle analysis, MECs were pulse-labeled with bromodeoxyuridine (BrdU) for 15 hours. Cells were then stained with propidium iodide (PI) and an antibody against BrdU and analyzed via flow cytometry as described [50]. For mammary tumor cell transplantation, 1 × 10^6^ tumor cells were transplanted into MFPs of NSG mice as previously described [25]. Four weeks post-transplantation, newly generated tumors were dissected and analyzed.

### LOH and methylation analysis

For LOH analysis, genomic DNA extracted from micro-dissected *p16^+/−^;Brca1^MGKO^* mammary tumor cells was analyzed with primers amplifying wild-type (WT) and knock-out (KO) allele of *p16* as previously described [12, 24]. For methylation analysis, genomic DNA from *p16^+/−^;Brca1^MGKO^* mammary tumors and tumor-free mammary tissues of the same mice were treated with bisulfide and analyzed for *p16* methylation with specific primers amplifying the unmethylated or methylated allele as described [12]. In addition, *p16* mRNA levels in primary *p16^+/−^;Brca1^MGKO^* mammary tumor cells treated with DAC at the indicated concentrations for 72 hours were analyzed by q-RT-PCR.

### Human breast cancer samples

Formalin fixed paraffin-embedded (FFPE) human breast cancer samples lacking patient-identifying information were obtained from the Tissue Bank Core Facility at the University of Miami. All samples obtained were non-treated invasive breast cancers with known ER status. The expression of *BRCA1* in tumors was determined by microdissection-based RNA extraction and Q-RT-PCR as we previously described [25].

### Statistical analysis

All data are presented as the mean ± SD for at least three repeated individual experiments for each group unless otherwise specified. Statistical analysis of mRNA expression, Ki67 positive cells, and tumor volume was performed using a two-tailed Student's *t-test*. Statistical analysis of tumor incidence was performed using a two-tailed Fisher's exact test. Statistical analysis of mammary tumor-free survival was performed using a Log-rank (Mantel-cox) test. *P <* 0.05 was considered statistically significant. Statistical analyses were conducted using Microsoft Excel and GraphPad Prism 5.

## SUPPLEMENTARY MATERIALS FIGURES AND TABLES


