# Supplementary material for: p16INK4a suppresses BRCA1-deficient mammary tumorigenesis
Source: Oncotarget. 2016 Nov 2;7(51):84496–507. doi: 10.18632/oncotarget.13015 (PMC5356676; doi:10.18632/oncotarget.13015)
Supplement: Supplementary file 1 [file oncotarget-07-84496-s001.pdf]

p16^{INK4a} suppresses BRCA1-deficient mammary tumorigenesis

Supplementary Materials

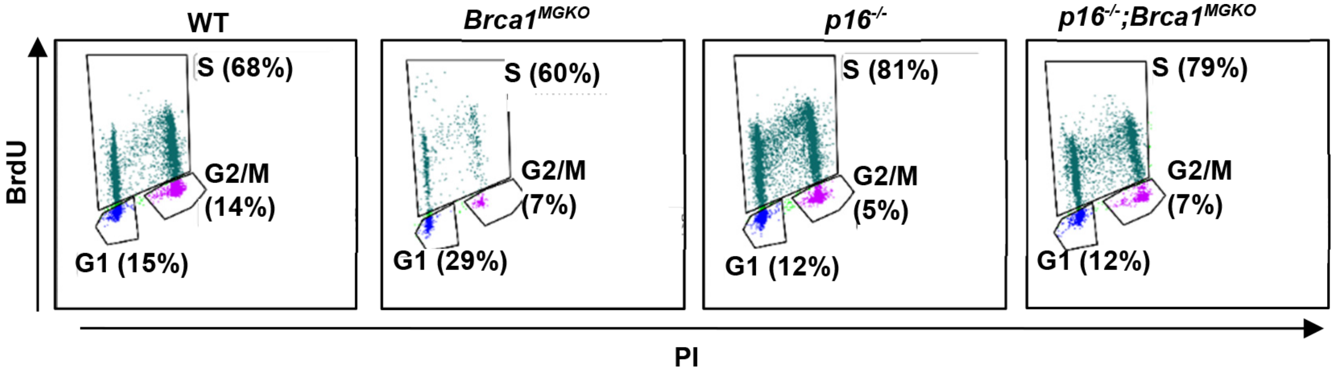

Supplementary Figure S1: Cell cycle analysis of primary MECs. Primary MECs of the indicated genotypes were pulse-labeled with bromodeoxyuridine (BrdU) for 15 hours and analyzed by FACS. Note the increase of *Brca1*^{MGKO} MECs in G1 and the decrease of cells in S phase relative to WT, and the decrease of *p16*$^{-/-}$;*Brca1*^{MGKO} MECs in G1 and increase of cells in S phase relative to *Brca1*^{MGKO}.

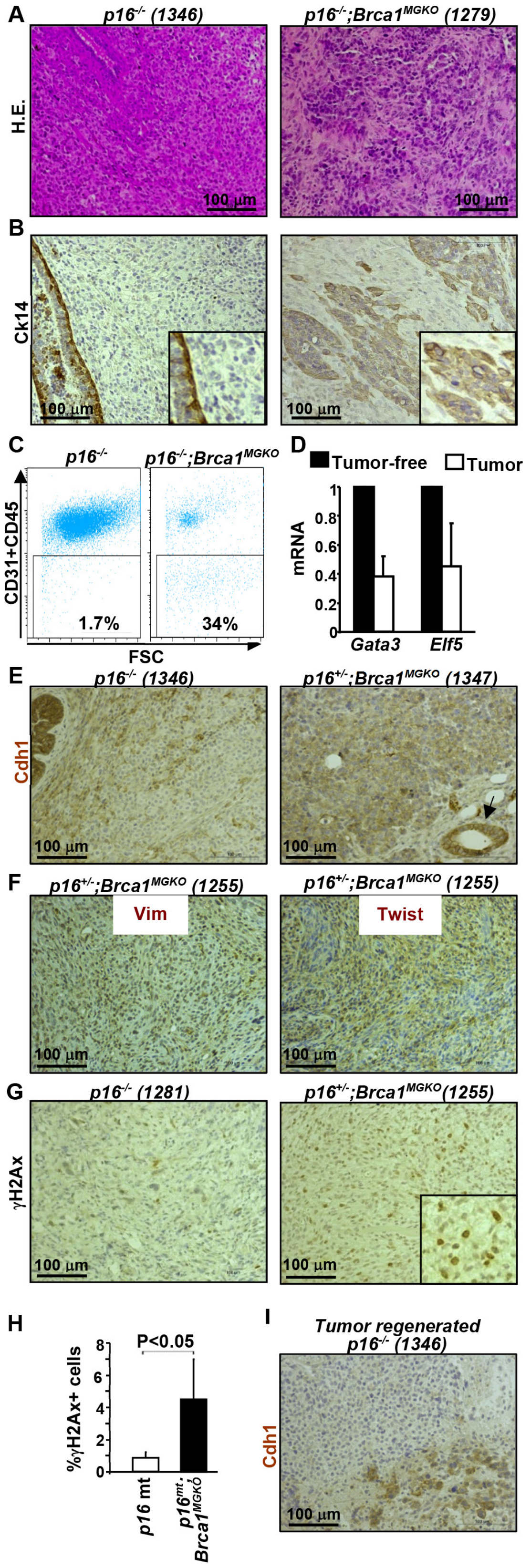

Supplementary Figure S2: Histological and molecular characterization of tumors developed in *p16$^{-/-}$* and *p16-/-;Brca1^{MGKO}* mice. (**A** and **B**) Representative H.&E. and immunostaining of Ck14 of primary tumors in mammary glands of the indicated genotypes. (**C**) Tumor cells from the mammaries of *p16$^{-/-}$* and *p16$^{-/-}$;Brca1^{MGKO}* mice were analyzed by flow cytometry for CD31 and CD45. (**D**) q-RT-PCR analysis for *p16^{mt};Brca1^{MGKO}* (*p16$^{+/-}$;Brca1^{MGKO}* and *p16$^{-/-}$;Brca1^{MGKO}*) mammary tumors and their corresponding tumor-free mammary tissues from same mice. Data are expressed as the mean ± SD of three mice. (**E**, **F**, **G**) Representative immunostaining of Cdh1(E), Vimentin (Vim), Twist (F), and γH2AX (G) for primary tumors in mammary glands. Note the reduced Cdh1 expression in *p16$^{+/-}$;Brca1^{MGKO}* tumor cells relative to that in luminal epithelial cells indicated. (**H**) The percentages of γH2AX-positive cells were calculated from >800 cells for each tumor. For p16 mutant (p16^{mt}) tumors, results from a sarcoma and two lymphomas were pooled. For *p16^{mt};Brca1^{MGKO}* tumors, results from a *p16$^{+/-}$;Brca1^{MGKO}* mammary tumor and two *p16$^{-/-}$;Brca1^{MGKO}* mammary tumors were pooled. Results represent the mean ± SD. (I) Representative immunostaining of *p16$^{-/-}$* tumors regenerated in mammary glands by primary tumor cells.

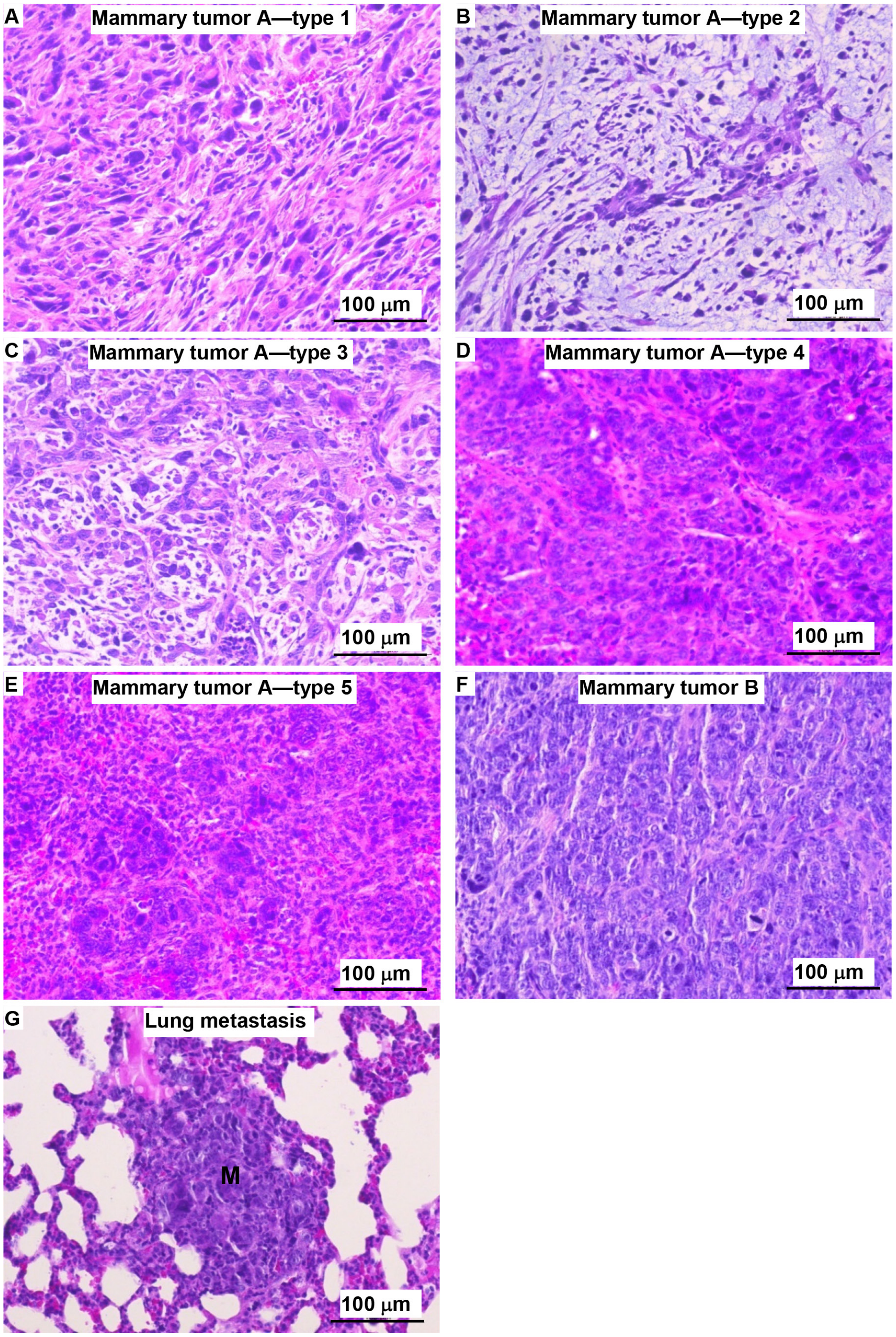

Supplementary Figure S3: Characterization of inter- and intra-tumor heterogeneity in mammary tumors. Representative H.E staining of a tumor developed in the right 3rd mammary gland (**A** - **E**), a tumor in the left 3rd mammary gland (**F**), and a tumor metastasized to lungs (**G**) in a *p16*$^{+/-}$;*Brca1*^{MGKO} mouse (*1509*). Note the different cell types in mammary tumors of this mouse.

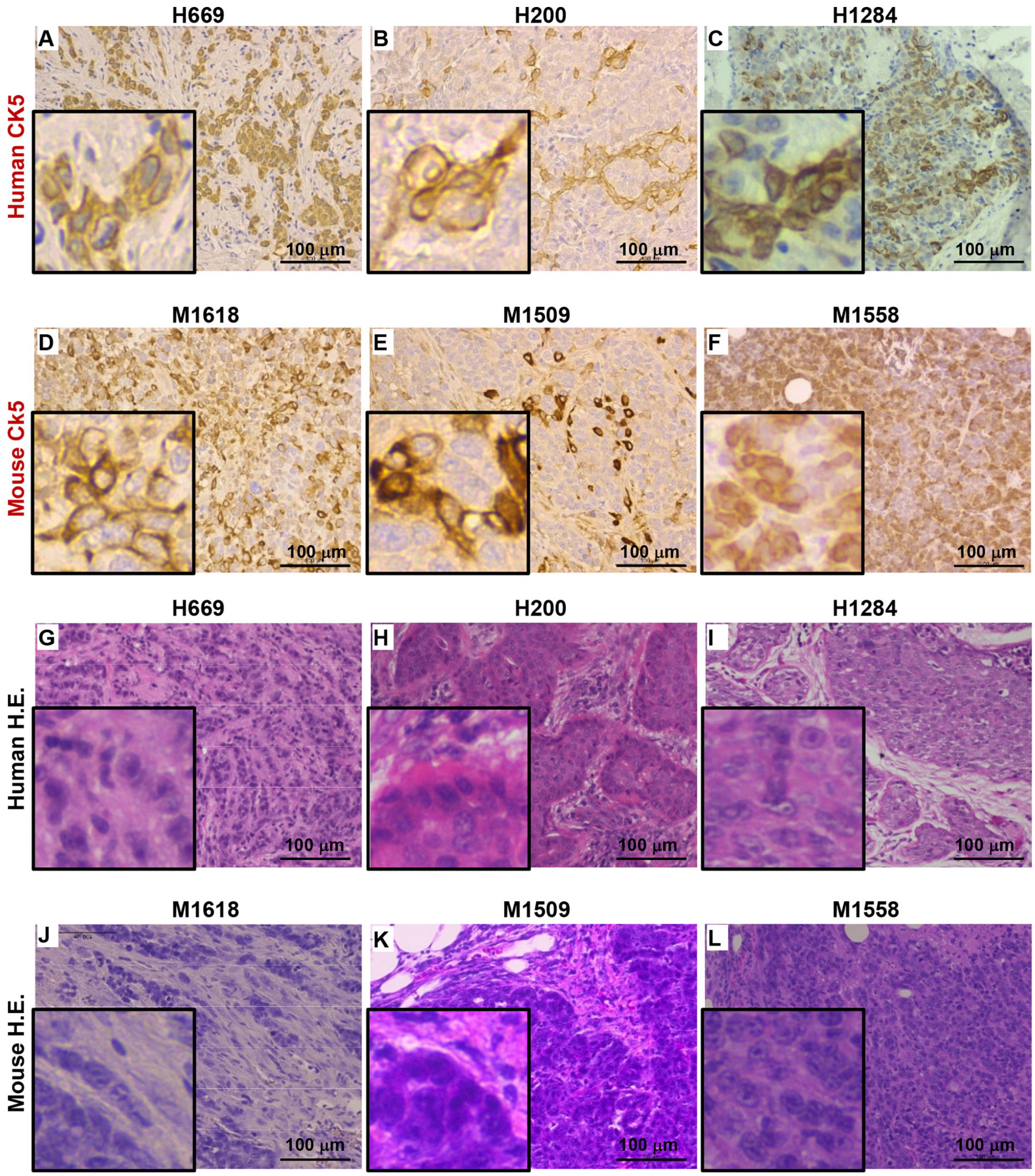

Supplementary Figure S4: Characterization of ER-negative human breast cancers and *p16;Brca1* mutant mouse mammary tumors. Representative CK5 (**A–F**) and H.E staining (**G–L**) of ER-negative human invasive breast cancers (A–C, G–I) and mammary tumors developed in *p16;Brca1* double-mutant mice (D–F, J–L) are shown. Note the similarity of tumor cell morphology and CK5 staining between human breast cancers and mouse mammary tumors.

Supplementary Table 1: Characterization of mammary tumors developed in mutant mice

Mouse ID	Age[a] (mo)	Genotype	Mammary tumor pathology	Ck5b	Ck14b	ERa	Lung metastasis
1255	18	$p16^{+/-}$;$Brca1^{MGKO}$	Adenocarcinoma with scirrhous pattern	++	+++	-	Yes
1279	12	$p16^{-/-}$;$Brca1^{MGKO}$	Adenocarcinoma	++	+++	-	
1288	16	$p16^{-/-}$;$Brca1^{MGKO}$	Adenocarcinoma	+	+++	-	
1347	19	$p16^{+/-}$;$Brca1^{MGKO}$	Adenocarcinoma with central necrosis	+	++	-	
1479	11	$p16^{-/-}$;$Brca1^{MGKO}$	A: Adenomyoepithelioma	++	+++	-	
			B: Adenocarcinoma with scirrhous pattern	++	+++		
1497	20	$p16^{+/-}$;$Brca1^{MGKO}$	Adenocarcinoma with cribriform pattern	++	+++	-	Yes
1509	18	$p16^{+/-}$;$Brca1^{MGKO}$	A: pleomorphic carcinoma	++	+++	-	
			B: Adenocarcinoma with central necrosis	++	+++		Yes
1558	20	$p16^{-/-}$;$Brca1^{MGKO}$	Adenocarcinoma with central necrosis	++	+++	-	Yes
1618	17	$p16^{-/-}$;$Brca1^{MGKO}$	A: pleomorphic carcinoma	++	+++	-	Yes
			B: Adenocarcinoma with central necrosis				

[a] mo, month

[b] -; <2%, +; 2-25%, ++; 25-50%, +++; 50-75%, ++++; >75%
